# Enabling Moisture and Interfacial Stability in Sulfide Solid Electrolytes via a Processable Organic Coating Strategy for High‐Voltage All‐Solid‐State Batteries

**DOI:** 10.1002/anie.9983580

**Published:** 2026-06-30

**Authors:** Lanting Qian, Cameron Dean, Ivan Kochetkov, Hengning Chen, Yangyang Huang, Linda Nazar

**Affiliations:** ^1^ Department of Chemistry Waterloo Institute of Nanotechnology University of Waterloo Waterloo Ontario Canada

**Keywords:** anode, argyrodite, cathode, chemical engineering, coating, electrolyte, ionic conductivity, materials science, moisture, sulfide

## Abstract

Sulfide solid electrolytes (SEs) are excellent candidates for solid‐state batteries (SSBs), but their extreme sensitivity to moisture and lack of oxidative stability with uncoated high‐voltage cathodes incur processing complexity and cost. Here, we present a simple and cost‐effective decanoate fatty‐acid (DA) coating strategy for argyrodite (Li_6_PS_5_Cl, LPSCl) that stabilizes it to exposure at 39% relative humidity for up to 2 h, while preserving its structure, ionic conductivity, and increasing its anodic stability. Cells employing 2 wt%‐coated LPSCl (DA–LPSCl) as the catholyte, with a bare NCM85 cathode and Li‐In anode, deliver a capacity of 175 mAh.g^−1^, and 96% capacity retention over 150 cycles at 0.2 C, while bare LPSCl retains only 61% capacity. Symmetric Li|DA–LPSCl|Li cells cycle for 1000 h, in contrast to bare LPSCl cells, which short‐circuit after ∼230 h. Moreover, full cells using a lithium metal anode with the DA–LPSCl SE showed remarkable performance compared to state‐of‐the‐art SSBs, retaining 81% of their capacity after 300 cycles at 0.2 C. High‐loading cells with areal capacities up to 3.2 mAh cm^−2^ are also demonstrated. This work showcases the potential of a low‐cost, processable, and flexible coating to address key limitations of sulfide SEs, advancing the commercial viability of SSBs.

## Graphical abstract

The solid electrolyte, argyrodite (Li_6_PS_5_Cl or LPSCl), “waxed” with a 25 nm coating of decanoic acid (DA; C_10_H_19_O_2_) is protected against hydrolysis at high relative humidity and maintains its ionic conductivity. DA–LPSCl exhibits enhanced stability at both NCM|DA–LPSCl and Li|DA–LPSCl interfaces: solid state batteries with a bare NCM85 cathode and Li anode show greatly improved performance compared to bare argyrodite at low‐moderate stack pressure.

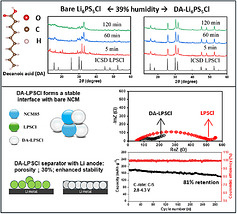

## 1 Introduction

Solid‐state batteries (SSBs) have garnered significant interest in the field of energy storage due to their potential to offer higher energy density and improved safety compared to conventional lithium‐ion batteries [1, 2, 3]. The advancement of high‐performance SSBs depends on several factors, such as the use of solid electrolytes (SEs) that possess favorable interfaces with the electrodes and exhibit high ionic conductivities at room temperature [4]. Among the state‐of‐the‐art SEs (including oxides, halides, and oxyhalides) [5, 6, 7], sulfide SEs such as Li_6_PS_5_Cl (LPSCl) stand out due to their high ionic conductivity [8, 9], compatibility with Li‐In anodes, soft mechanical properties, and composition of earth‐abundant elements.

However, the widespread adoption of sulfide SEs is impeded by several critical challenges, one of which is their sensitivity to moisture [10]. When exposed to water vapor, sulfide SEs rapidly hydrolyze, releasing toxic H_2_S gas and experience a drastic loss in ionic conductivity. Consequently, these materials require stringent handling conditions—typically within argon‐filled gloveboxes maintained at dew points as low as −80°C in academic laboratories [11, 12]. In industrial settings, processing of these SEs must occur in dry rooms with dew points below −60°C, substantially increasing operational costs compared to the −40°C conditions standard in conventional lithium‐ion battery manufacturing [9, 13]. Moreover, on the cathode side, sulfide catholytes undergo oxidative decomposition above 2.5 V versus Li^+^/Li, leading to significant capacity fade when paired with high‐voltage cathodes operating up to 4.3 V. This degradation is primarily attributed to the formation and accumulation of interfacial decomposition products—such as phosphorus‐ and sulfur‐containing oxides (PO_x_ and SO_x_)—which increase interfacial resistance and hinder lithium‐ion transport across the cathode–catholyte interface (CEI) [14, 15]. To address this issue, considerable effort has been made to apply protective layers onto the cathode. To date, inorganic coatings such as Al_2_O_3_ [16], ZrO_2_ [17], Li_2_SiO_3_ [18], LiNbO_3_ [19], and Li_2_ZrO_3_ [20] have proven effective. However, many of these inorganic coatings require complex fabrication steps, making them less applicable in commercial settings. On the anode side, LPSCl forms a quasi‐stable and passivating interphase composed of Li_2_S, Li_3_P, and LiX (X = Cl, Br) [21, 22]. Although this interphase can partially suppress interfacial reactions, dendrite growth remains a significant challenge unless the electrolyte achieves a relative density exceeding 95% [21, 23]. Strategies such as metal or anion substitution in LPSCl, or applying a coating interlayer on top of the Li anode have been reported to improve cell cycling stability [24, 25, 26, 27, 28]. However, the use of protective layers or modifications on both the cathode and anode sides substantially increases the complexity and costs of the SSB. Compared with the extensive literature on cathode coatings, surface‐engineering strategies that directly modify sulfide SEs—particularly LPSCl—remain relatively limited. To date, only a relatively small number of studies have reported coating or surface‐modification approaches for LPSCl, including inorganic layers [29, 30, 31], organic layers [11, 32, 33, 34], carbon‐based coatings [35], and polymer layers [36, 37] primarily aimed at improving air stability or interfacial compatibility.

In this work, we address these key challenges associated with sulfide SEs by employing a simple and scalable strategy to engineer an nanometric organic fatty‐acid coating (∼25 nm) on LPSCl particles. The process is carried out under simple conditions and does not require high‐temperature sintering, making it low‐cost and suitable for mass production. The decanoate (C_10_H_19_O_2_; i.e., DA) coating binds strongly to the LPSCl surface and greatly increases its anodic stability; it remains intact during composite cathode fabrication, and is chemically compatible with LPSCl. Notably, DA‐coated LPSCl (DA–LPSCl) demonstrates exceptional air stability, retaining an ionic conductivity above 1 mS cm^−^^1^ even after exposure to 39% relative humidity for up to 2 h, whereas bare LPSCl degrades almost instantly. In all‐solid‐state battery (ASSB) configurations using a Li‐In anode, the DA–LPSCl SE enables excellent cycling stability, retaining 90% of the initial capacity after 300 cycles, whereas uncoated LPSCl preserves only 61%. Furthermore, Li|Li symmetric cells employing DA–LPSCl exhibit stable plating/stripping for 1000 h at 1 mAh cm^−^^2^ with negligible polarization growth, while bare LPSCl short‐circuits after ∼200 h. We correlate this to the higher density of the DA–LPSCl separator (30% reduction in porosity), and more uniform contact with Li metal owing to the soft nature of the decanoate coating. Full cells pairing a lithium metal anode with DA–LPSCl delivered high areal capacity and maintained 81% of their initial capacity over 300 cycles at 0.2 C, while high‐loading cells (21 mg cm^−2^) provide areal capacities up to 3.2 mAh cm^2^. These results highlight the effectiveness of a low‐cost, scalable, and conformal surface coating in addressing the intrinsic limitations of sulfide electrolytes, providing a promising pathway toward the practical realization of high‐performance ASSBs.

## 2 Results and Discussion

### 2.1 Coating Morphology

**FIGURE 1:**
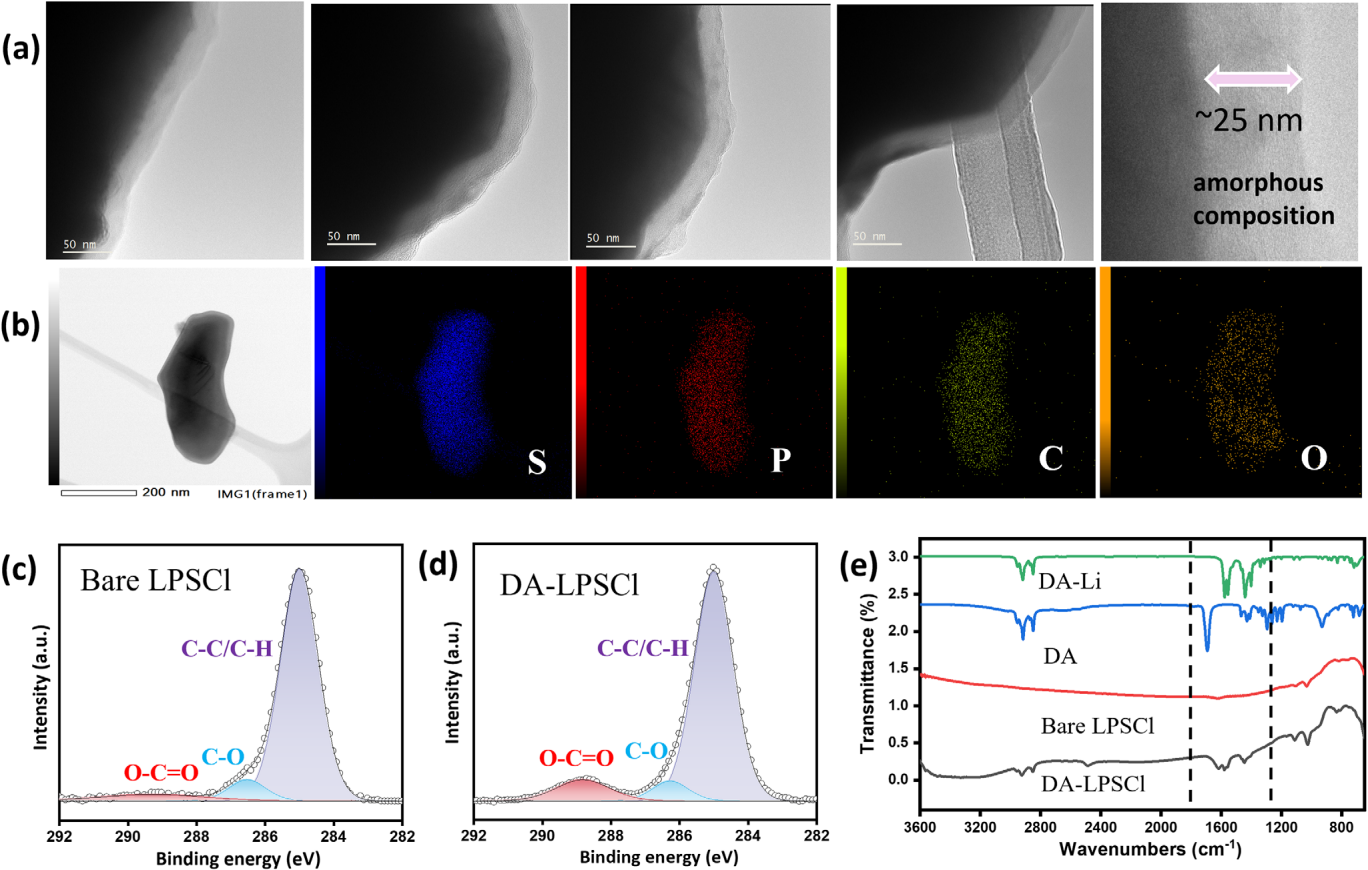
Characterization of DA‐coated LPSCl. (a) Cryo‐TEM images of coated LPSCl; (b) EDX maps of DA–LPSCl; C 1s XPS spectra of (c) bare LPSCl and (d) DA–LPSCl; (e) FTIR spectra of control samples, bare LPSCl and DA–LPSCl.

DA was deposited onto the LPSCl particles using a solution‐assisted method (see Supporting Information for details). Scanning electron microscopy (SEM) (Figure S1) shows that the DA–LPSCl particles exhibit irregular shapes and a broad size distribution like bare LPSCl, with no obvious aggregation after coating. Powder x‐ray diffraction (PXRD) further confirms that the bulk structure remains unchanged after coating (Figure S2), as expected. Cryo‐transmission electron microscopy (CR‐TEM) images revealed a conformal and amorphous coating that is ∼25 nm thick on the particle surface of LPSCl (Figure 1a). This was confirmed by energy dispersive x‐ray spectroscopy (EDX) mapping of the DA–LPSCl, showing the coating is comprised of C and O elements that are uniformly distributed on the surface of the LPSCl (Figure 1b). C 1*s* x‐ray photoelectron spectroscopy (XPS) spectra revealed a significant increase in the contribution of the carboxyl functional group (O─C═O) following the coating process (Figure 1d), relative to bare LPSCl (Figure 1c), confirming successful deposition of the coating onto the SE surface. This observation was further corroborated by Fourier‐transform infrared (FTIR) spectroscopy (Figure 1e). Compared to bare LPSCl, the FTIR spectrum of DA–LPSCl shows new peaks at about 1600–1350 cm^−^^1^ and at about 3000–2800 cm^−^^1^. The absorption bands in those regions are assigned to C─H stretching vibrations of methylene (─CH_2_─CH_2_─) groups, while bands between 1700 and 1350 cm^−^^1^ are attributed to C═O stretching vibrations. These vibrational modes also align with characteristic features for DA–Li compounds, suggesting the formation of ‐COOLi on the LPSCl surface, which we have shown to be permeable to Li^+^‐ions on cathode surfaces [38].

### 2.2 Chemical Compatibility and Interaction of the Coating With LPSCl

**FIGURE 2:**
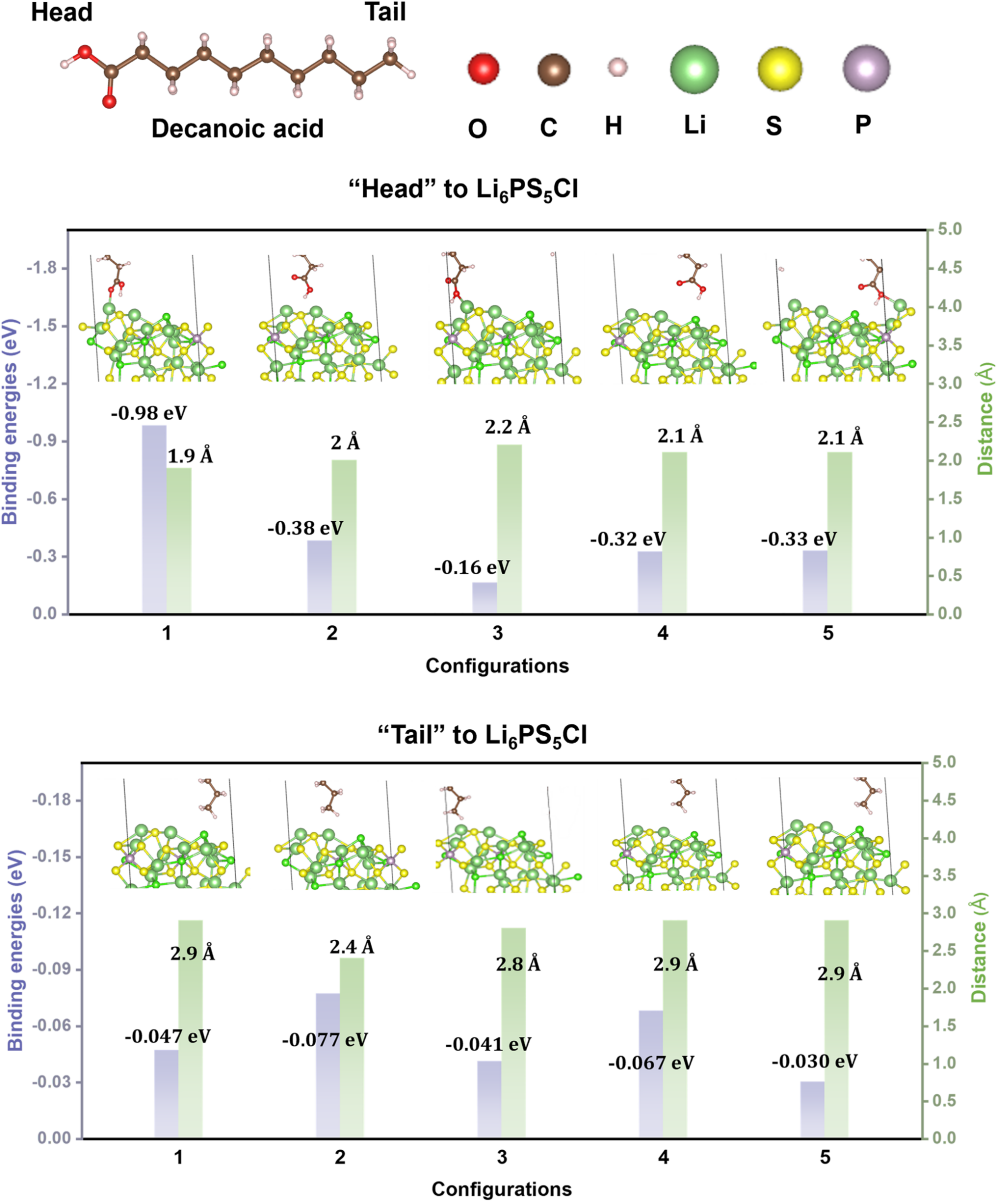
Comparison of DFT‐calculated binding energies (blue), distances (green) and adsorption geometries of DA on the LPSCl (010) surface via the polar head and nonpolar tail.

Chemical and mechanical adhesion are the two principal mechanisms by which a coating can bind to a substrate surface. In the context of SSB fabrication, chemical binding is advantageous, as it facilitates strong interfacial contact of the coating and the SE during preparation of the cathode active material (CAM) composite. Insufficient binding may lead to coating delamination or mechanical degradation during mixing. In our previous work [28], we demonstrated the successful application of a robust DA coating onto the NCM surface via an acid–base reaction with surface residuals such as carbonates and hydroxides, resulting in the formation of lithium decanoate [24]. We hypothesized that LPSCl may also undergo a surface reaction with the carboxylate functional groups in DA, leading to the formation of a chemically bonded and stable coating layer. To investigate this premise, we performed density functional theory (DFT) calculations to evaluate the interactions of DA molecules on the LPSCl surface (Figure 2). The (010) facet of LPSCl was selected for analysis, as it was found to exhibit the lowest surface energy (Figure S3, Table S2). To understand how DA molecules interact with the LPSCl surface, the ground‐state energy of common DA molecule conformations was determined (Figure S4). A linear conformation of DA was found to be the most stable (Table S1). In our analysis, we classified DA adsorption configurations into two main categories: (1) polar‐head adsorption, where the ─COOH functional group faces the LPSCl surface, and (2) nonpolar‐tail adsorption, where the alkyl chain interacts with the surface (Figure 2). Configurations in which the DA molecule is parallel to the surface were excluded due to the high computational cost required to isolate the full‐length carbon chain from its periodic image. Moreover, such parallel configurations are considered physically unlikely, as they primarily consist of weak van der Waals interactions and impose an entropic penalty by restricting alkyl‐chain conformational freedom. Multiple adsorption geometries were explored within each category to identify the most energetically favorable configurations (Figures 2, S5, and S6) and to gain insight into the adsorption/binding behavior of DA on LPSCl. The binding energy (*E*_bind_) was calculated using the following expression:

(1) $$\begin{array}{cc} & \mathrm{Binding}\mathrm{Energy}=E\left(\mathrm{deposited}\;\mathrm{DA}\right)-\;E\left(\mathrm{surface}\right)\\ & -\;E\left(DA\;\mathrm{molecule}\right) \end{array}$$

The resulting binding energies are summarized in Figure 2, and span from −0.16 to −0.98 eV, indicating favorable interactions between DA and the LPSCl surface. The strongest binding (−0.98 eV) occurs when the carboxylic acid headgroup of DA coordinates to the surface Li^+^‐ions, forming Li–O bonds with distances of ∼1.9 Å. This value is comparable to typical Li─OH bond lengths (1.9–1.95 Å)—suggesting formation of lithium decanoate (─COOLi) via release of HCl. In contrast, when the nonpolar alkyl tail faces the LPSCl surface, binding energies are significantly weaker (e.g., 0.077 eV), with adsorption distances exceeding 2.5 Å, indicative of only van der Waals interactions. These results confirm that DA preferentially adsorbs onto the LPSCl surface via its polar headgroup through Li─O bonding, forming a ─COOLi functional group that facilitates Li^+^‐ion conduction [39, 40]. This corroborates the FTIR results. Full computational details are provided in the Supporting Information.

### 2.3 Moisture Stability

**FIGURE 3:**
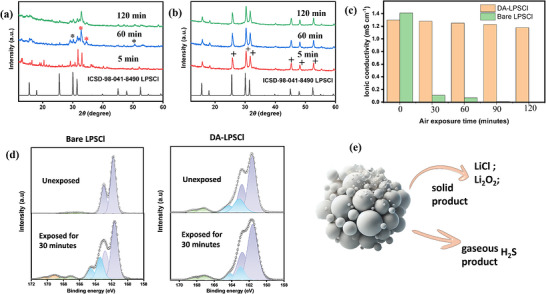
Effectiveness of the coating against moisture. X‐ray diffraction patterns of (a) bare LPSCl and (b) DA–LPSCl monitored over time. The black * represent LiCl reflections, the red * denotes the Li_2_O_2_ reflections, and + denotes the LPSCl reflections; (c) comparison of ionic conductivities of bare and DA–LPSCl as a function of exposure time in humid air; XPS spectra of (d) bare LPSCl and (e) DA‐coated LPSCl before and after exposure to moisture; (e) schematic illustration of the degradation of LPSCl upon moisture exposure.

The air stability of bare LPSCl and DA–LPSCl was evaluated by exposing them to a typical indoor relative humidity (∼39%) for different durations. PXRD and XPS were employed to monitor the degradation process. Bare LPSCl (Figure 3a) exhibited rapid changes in its diffraction pattern, marked by the immediate formation of Li_2_O_2_ and LiCl. In contrast, DA–LPSCl (Figure 3b) showed no discernible changes in its x‐ray diffraction (XRD) pattern even after 2 h of exposure, suggesting that the coating effectively suppresses moisture‐induced decomposition of the bulk structure. Figure 3c shows ionic conductivities after exposing LPSCl to moisture. While the bare LPSCl shows an immediate decrease in ionic conductivity, which is continuous for up to 2 h, the DA–LPSCl shows only a slight decrease after 2 h. These changes in ionic conductivity are consistent with the structural degradation of the bare LPSCl and integrity of DA–LPSCl monitored using XRD.

Figure 3d presents the S 2*p* XPS spectra for bare LPSCl and DA–LPSCl, respectively, after 30 min of exposure under humid air. The S 2*p*_3/2_ peak at 161.6 eV corresponds to sulfide in the PS_4_^3−^ tetrahedra characteristic of the argyrodite structure. The contribution at ∼163.2 eV is attributed to the S 2*p*_3/2_ peak of elemental sulfur generated from oxidation of the sulfide [41]. This peak increases markedly for bare LPSCl after 30 min of exposure. In contrast, the oxidation‐related signal in DA–LPSCl remains nearly unchanged before and after exposure, demonstrating that the DA coating effectively mitigates the reaction between LPSCl and atmospheric water. The peaks at 166.6 and 168 eV correspond to thiosulfate and polythiosulfate species, respectively. The initial presence of elemental sulfur and thiosulfate species in DA–LPSCl likely arises from surface oxidation induced by interactions between the carboxyl (─COOH) group of decanoic acid and the LPSCl surface. Importantly, the coexistence of elemental sulfur and thiosulfate species likely plays a role by forming a passivating interphase that limits further hydrolysis of LPSCl. The XPS spectra corroborate reports on the degradation mechanism of LPSCl, in which substantial oxidized sulfur species are formed [35]. Figure 3e illustrates a schematic representation of bare LPSCl upon moisture exposure. The P 2*p* spectra (Figure S7) of DA–LPSCl exhibit no discernible signal, indicating that the surface is predominantly composed of oxidized sulfur species (e.g., sulfate/thiosulfate) together with carbon chains of decanoic acid. This surface composition likely plays a critical role in enhancing resistance to air exposure. However, given the limited probe depth of XPS, the possibility of subsurface chemical products, such as phosphates, cannot be excluded. In addition to this chemically passivating layer, the decanoic acid coating itself likely contributes to the improved air stability through a physical barrier effect. Decanoic acid comprises a long hydrophobic alkyl (C_10_) chain, which reduces the accessibility and diffusion of moisture to the underlying sulfide surface [11, 34, 35, 42, 43]. The organic layer therefore acts as a protective interface that further suppresses direct contact between water molecules and the highly moisture‐sensitive thiophosphate moiety. Considering these factors, the enhanced environmental stability of DA‐coated LPSCl is most reasonably attributed to a synergistic effect between the formation of a thin oxidized interphase and the moisture‐blocking function of the hydrophobic carbon chain. Figure 3e illustrates a schematic representation of bare LPSCl upon moisture exposure. To place our results in context, a comparison of representative surface‐modified LPSCl systems reported in the literature is summarized in Table S4. Despite the wide variation in testing conditions, including air exposure time and relative humidity, our material demonstrates performance comparable to or better than the best reported cases.

### 2.4 Electrochemical Performance

**FIGURE 4:**
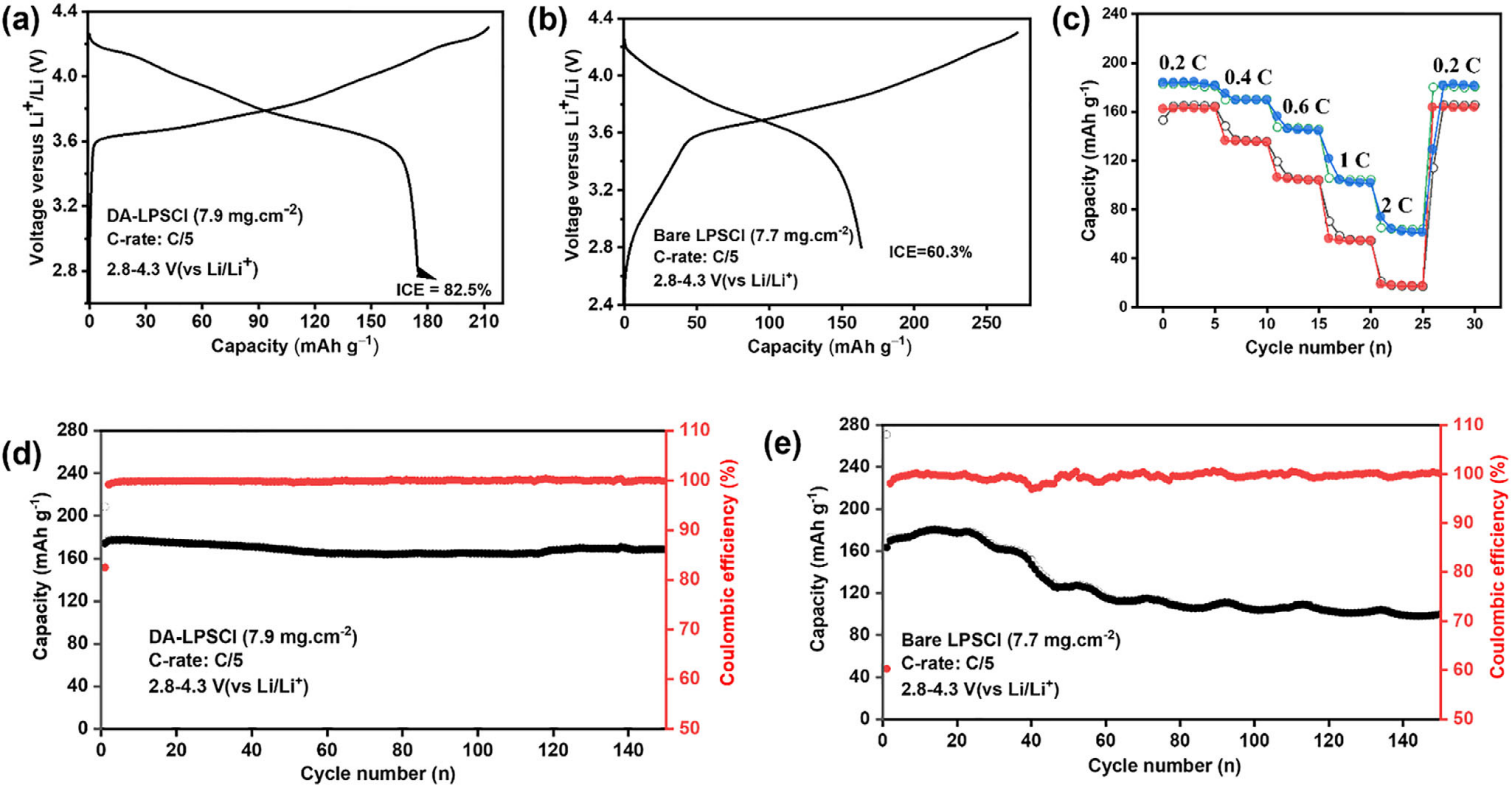
Electrochemical performance of bare LPSCl and DA–LPSCl SEs using a cathode composite comprised of bare NCM85 mixed with the SE, and a Li‐In anode. Initial charge/discharge profiles of SSBs using (a) DA–LPSCl and (b) bare LPSCl as the SE in a 2.8–4.3 V window; (c) Rate performance of SSB batteries assembled with bare LPSCl and DA–LPSCl; Cycling battery performance of (d) DA–LPSCl and (e) bare LPSCl.

ASSBs were assembled using DA–LPSCl (2 wt% DA) or bare LPSCl as both the catholyte and separator, with Li–In as the anode. The ionic conductivity significantly decreases as the DA content increases beyond 2 wt% (Table S3). Consistently, ASSBs assembled with 5 wt% DA–LPSCl (Figure S8) exhibit significantly lower discharge capacity, showing that 2 wt% DA–LPSCl is the optimal composition. The first charge/discharge cycle was used to evaluate the initial Coulombic efficiency (CE) and the effectiveness of the DA coating. Figure 4a presents the initial charge–discharge profiles of LPSCl and DA–LPSCl cells cycled at a rate of 0.2 C in a voltage window of 2.8–4.3 V versus Li^+^/Li. DA–LPSCl delivers a slightly higher initial discharge capacity (175 vs. 163 mAh g^−^^1^) and significantly improved initial CE (83% vs. 60%), indicating suppressed interfacial reactivity between NCM85 and LPSCl. The ASSB with bare LPSCl displayed a pronounced sloping region below 3.5 V during the initial charge (Figure 4b), characteristic of LPSCl oxidation. This feature is absent in the voltage profile of the DA–LPSCl cell, confirming that the coating effectively mitigates electrolyte oxidation. The rate performance of coated and uncoated LPSCl with NCM85 in ASSBs is shown in Figure 4c. The coated LPSCl delivers high discharge capacities of 183 mAh g^−^^1^ at 0.2 C and 104 mAh g^−^^1^ at 1 C, significantly outperforming its uncoated counterpart, which achieves only 162 and 55 mAh g^−^^1^ at the same respective rates. The inferior performance of the uncoated cell arises from LPSCl oxidation during cycling, producing insulating PO_x_ and SO_x_ species at the interface between the SE and NCM85, which increase the interfacial resistance and hinder performance at high C‐rates. Figure 4d,e compares the long‐term cycling performance of cells with bare or DA–LPSCl at a similar loading. The DA–LPSCl cell maintains a high capacity of 168 mAh g^−^^1^ after 150 cycles at 0.2 C, corresponding to 96% capacity retention. In contrast, the bare LPSCl cell retains only 61% of its initial capacity, delivering just 99 mAh g^−^^1^ after 150 cycles. The performance of DA–LPSCl is comparable or superior to that of cells employing LPSCl with established coating materials on NCM, such as LiNbO_3_ [44], Li_2_ZrO_3_ [45], Li_3_BO_3_‐Li_2_CO_3_ [46], and Li_2_HfO_3_/HfO_2_ [47]. For instance, at a similar loading, LiNbO_3_‐coated cathode ASSBs displayed a capacity retention of 84 % after 75 cycles [33], while Li_2_HfO_3_/HfO_2_ coatings provided retention of 81 % after 200 cycles (but at 45°C) [44].

Cells cycled at 0.2 C up to a higher upper cutoff voltage of 4.6 V also demonstrated excellent performance, retaining 91% of their initial capacity after 110 cycles, further highlighting the effectiveness of the coating for high‐voltage applications (Figure S9). Although our cells demonstrate strong performance at a loading of ∼7 mg cm^−2^, areal capacities exceeding 3 mAh cm^−2^ are required to compete with commercial LIBs. Notably, Figure S9 presents a high‐loading cell (21 mg cm^−2^) that delivered an initial areal capacity of ∼3.2 mAh cm^−2^, and retained 81% of its capacity after 150 cycles at 0.2 C.

### 2.5 Origin of Enhanced Performance

**FIGURE 5:**
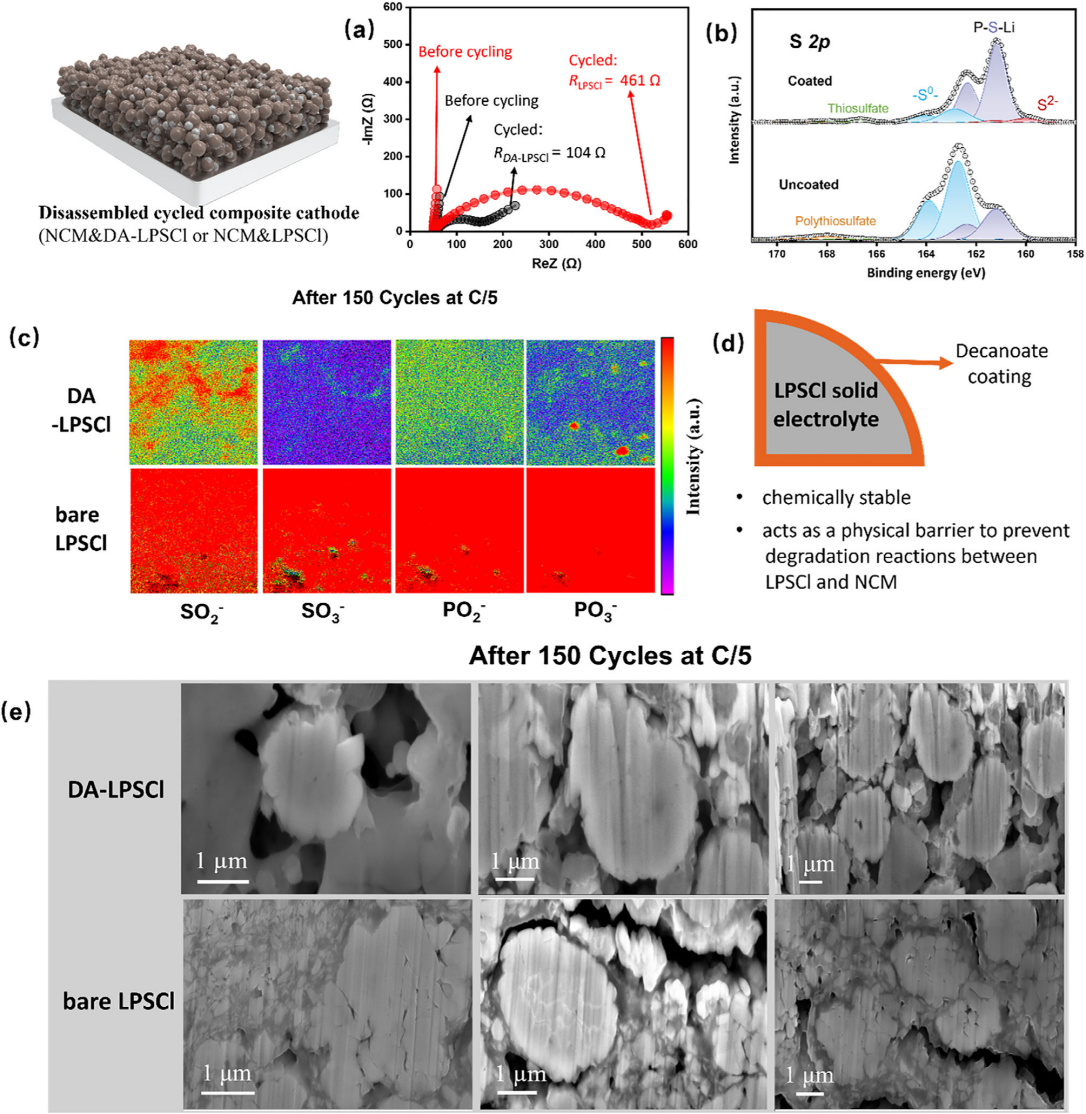
Origin of enhanced performance of SSBs fabricated with DA‐coated LPSCl compared to bare LPSCl. (a) Nyquist plots of cells assembled with bare LPSCl and DA–LPSCl before and after cycling. Cycled cells are fitted with a transmission line model; (b) S 2*p* XPS spectra of bare and DA–LPSCl after cycling; (c) ToF‐SIMS heat maps of SO_x_ and PO_x_ signals after 150 cycles; (d) schematic of the protection mechanism of DA‐coated LPSCl; (e) FIB cross‐section images of bare LPSCl and DA–LPSCl after 150 cycles at 0.2 C.

Electrochemical impedance spectroscopy (EIS) was used to evaluate impedance growth from SE–NCM85 decomposition in bare and DA–LPSCl cells before and after 150 cycles at 0.2 C (Figure 5a). The impedance spectra consist of a bulk SE resistance (*R*_bulk_) and a cathode resistance (*R*_cathode_). Prior to cycling, the cells show similar bulk resistance compared to that of the cycled cells. *R*_cathode_ values for the coated and uncoated cathodes were determined using a transmission line model (Figure S7) with blocking boundary conditions to fit the experimental data, following the procedure described in our previous study [15]. Upon cycling for 150 cycles at 0.2 C, both cells show larger resistance as a result of the decomposed products, but their resistance growth is much less in the cell using DA–LPSCl. The *R*_cathode_ of the cell with bare LPSCl (461 Ω) is approximately 4.4 times that of the cell with the DA–LPSCl (104 Ω). To better understand this behavior, we carried out cyclic voltammetry (CV) (Figure S11) to highlight the beneficial role of the DA coating in mitigating oxidative decomposition of the LPSCl. Bare LPSCl exhibits a pronounced anodic current onset at ∼2.5 V, indicative of significant electrolyte decomposition. In contrast, DA–LPSCl shows a markedly suppressed current response over the same potential range. This behavior is attributed to the electronically insulating nature of the DA layer, which limits electron transfer and thereby mitigates electrolyte oxidation. Overall, the CV results demonstrate that the DA coating enhances the practical oxidative stability of LPSCl.

To correlate battery performance with interfacial decomposition products, XPS was conducted on cycled composite cathodes from bare and DA–LPSCl cells (Figure 5b). The S 2*p* XPS spectrum of DA–LPSCl was fitted with three contributions defined according to their 2*p*_3/2_ binding energies: The signal observed at 160.2 eV is attributed to the “free” S^2−^ ions in LPSCl, and the signal at ∼161.1 eV is assigned to the PS_4_^3−^ tetrahedra of argyrodite [7]. The peak at ∼163.1 eV corresponds to elemental sulfur formed via oxidation of LPSCl. This peak is suppressed in the DA–LPSCl spectra but appears with significant intensity in the bare LPSCl composite, confirming that the DA coating effectively mitigates the oxidative decomposition of LPSCl.

Time‐of‐flight secondary ion mass spectrometry (ToF‐SIMS) was used to achieve a detection sensitivity several orders of magnitude higher than XPS and to quantify molecular fragments. A relatively large analysis area (200 × 200 µm), encompassing numerous SE–NCM85 interfaces and multiple NCM85 particles (*D*_50_ ≈ 5 µm), was selected to minimize sampling bias. All secondary‐ion signals were normalized to the total ion count, and intensities were extracted from the corresponding normalized images. The heat maps (Figure 5c) revealed extensive formation of SO_2_^−^, SO_3_^−^, PO_2_^−^, and PO_3_^−^ fragments in the bare LPSCl composite cathode, whereas these signals were much less intense in the DA–LPSCl composite cathode. This continued degradation between the NCM and LPSCl is driven by the diffusion of oxygen to the surface of the NCM, as previously documented [48, 49]. A schematic illustrating the protective influence of the DA‐coated LPSCl is presented in Figure 5d.

Manthiram and co‐workers [50] discussed the influence of surface/interface chemical reactivities in driving particle‐cracking behavior in high‐Ni NCM cathodes in their perspective article, challenging the prevailing view that such cracking arises solely from anisotropic lattice strain. The authors suggested that the surface reactivity—especially surface oxygen loss triggered by electrolyte‐cathode reactions at high states of charge—drives particle cracking rather than purely mechanical stress from lattice volume changes. To investigate this further, we performed focused‐ion beam (FIB)‐SEM to examine the morphological changes in the composite cathode cross sections after cycling. Cross‐sectional images (Figure 5e) reveal intragranular cracking in both bare LPSCl and DA–LPSCl composite cathodes. Additional FIB‐SEM images with corresponding EDX mapping supporting this observation are provided in Figures S8 and S9. The observed intragranular cracks typically initiate in the particle core and propagate radially outward, consistent with previous reports [15, 51]. Notably, however, the extent of fracture is markedly reduced in the DA–LPSCl cathode. The volume changes inherent to NCM cathodes are unlikely to be completely suppressed by such thin surface layers. Accordingly, the reduced cracking observed in the DA–LPSCl cathodes cannot be rationalized by bulk mechanical stabilization alone. Instead, we attribute such differences in behavior to interfacial degradation processes. The formation of insulating SO_x_ and PO_x_ species at the interface can generate localized regions of high current density, imposing additional mechanical stress on NCM particles. Furthermore, sulfide SEs under high‐voltage oxidative conditions can release gaseous byproducts (e.g., sulfur‐containing gases), which further exacerbate mechanical stress and promote fracture during cycling. Taken together, these results demonstrate that particle cracking in sulfide‐based ASSBs should be viewed primarily as a symptom of interfacial chemical reactivity rather than a direct consequence of reversible lattice volume changes in NCM cathodes. This conclusion represents a central mechanistic insight of the present work and is consistent with recent analyses by Park et al. [52]. Our results demonstrate that the degradation of LiNi_1__−_ *_x_*_−_ *ᵧ*Co*_x_* Mn*ᵧ*O_2_ (0.3 < 1 − *x* − *y* ≤ 0.8) in sulfide‐based ASSBs is more affected by irreversible decomposition of LPSCl at the NCM–LPSCl interface, rather than by bulk structural characteristics such as reversible lattice volume change. Corroborating evidence from EIS, ToF‐SIMS, and XPS, together with morphological insights from FIB‐SEM, confirms that the DA coating suppresses oxidative degradation of LPSCl, thereby mitigating particle cracking and ultimately enhancing battery performance.

### 2.6 Symmetric and Full‐Cell Behavior With Li Metal

**FIGURE 6:**
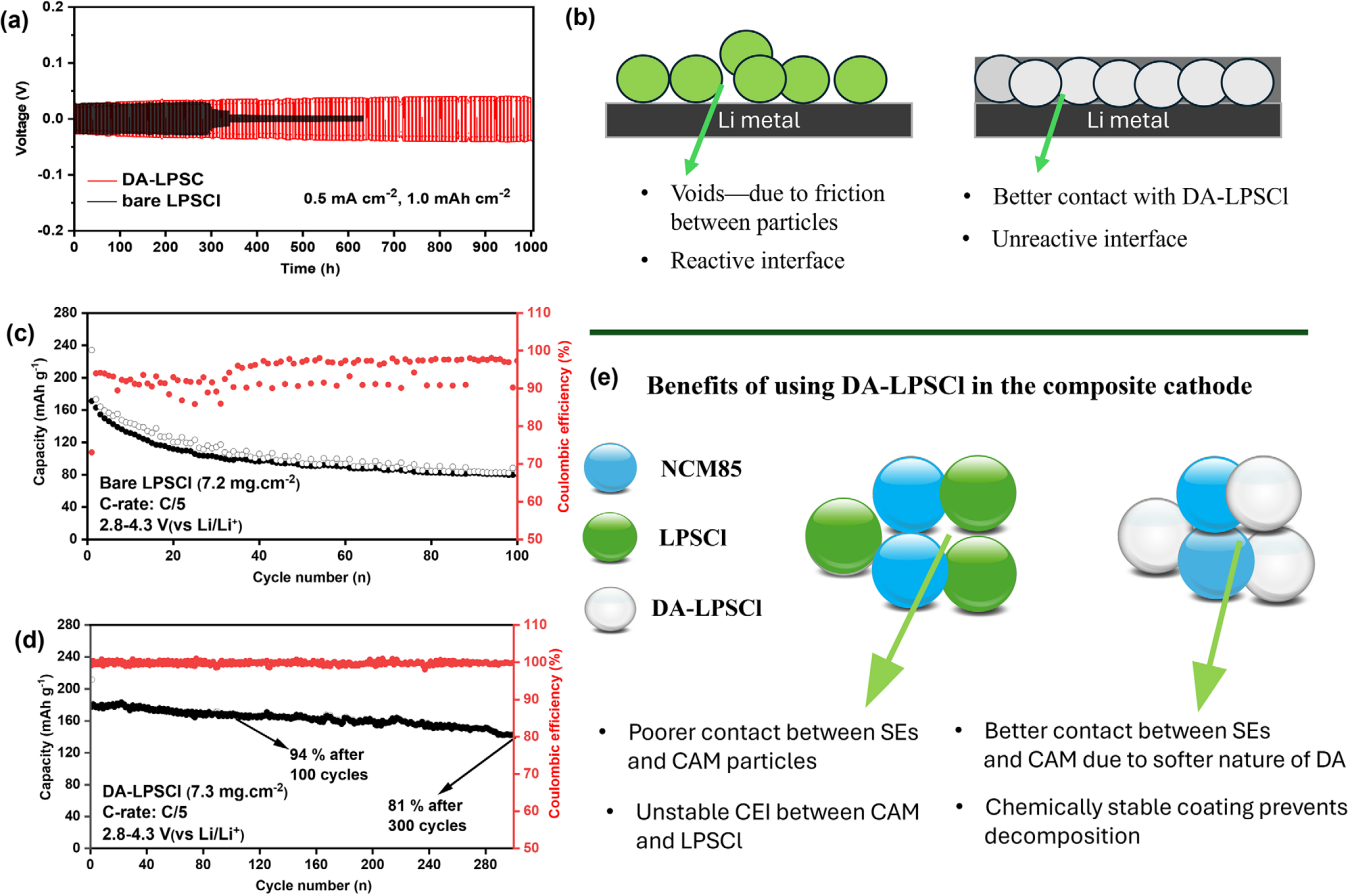
Electrochemical performance of symmetric cells using bare LPSCl or DA–LPSCl. (a) Comparison of Li‐Li symmetric cells assembled with DA–LPSCl and bare LPSCl; (b) schematic illustration of the role of DA in stabilizing the Li‖Li interface; full SSBs with Li metal anodes and NCM85 cathodes using (c) bare LPSCl or (d) DA–LPSCl run at 35 MPa; (e) schematic illustration of the beneficial role of DA in the composite cathode.

Prior studies have shown that a quasi‐stable Li|LPSCl interface can be achieved, yet dendrite penetration remains a critical challenge [21, 22]. To probe interfacial stability, Li‖Li symmetric cells were cycled at a current density of 0.5 mA cm^−^^2^ with an areal capacity of 1.0 mAh cm^−^^2^ (Figure 6a) using a custom‐made cell to ensure uniform uniaxial pressure. Although both DA–LPSCl and bare LPSCl can initially form a quasi‐stable interface with Li metal, the bare LPSCl symmetric cell short‐circuited earlier. The DA–LPSCl cell sustained stable cycling for up to 1000 h, with only a slight increase in polarization voltage, whereas the bare LPSCl cell failed after ∼230 h due to dendrite growth. Notably, the DA–LPSCl symmetric cell achieved performance comparable to that of a Li/LiPON/LPSCl/LiPON/Li configuration (using LiPON as an interlayer) under the same current density, but with an even higher areal capacity [53].

This enhanced stability is attributed to the soft nature of the DA coating, which promotes intimate wetting and uniform interfacial contact with Li, in contrast to the stiffer LPSCl particles. In principle, long‐chain fatty acids form molecular layers that are governed by weak van der Waals interactions and therefore exhibit considerably lower stiffness than inorganic sulfide electrolytes such as LPSCl. Their elastic modulus (∼0.1–2 GPa) is substantially lower than that of LPSCl (∼30 GPa), and their low melting point (∼31–32°C) can be directly correlated to their low stiffness (Young's modulus), as materials with higher melting points are stiffer, while those with lower melting points are more ductile. As a result, fatty acids are commonly incorporated into polymer composites to enhance softness [54, 55, 56, 57]. This mechanical compliance enables the DA coating to deform under stack pressure, improving interfacial conformability with Li metal, reducing void formation, and accommodating stresses during repeated plating/stripping, thereby contributing to enhanced interfacial stability. In the bare LPSCl cell, we propose that poor interfacial contact promotes non‐uniform current distribution and localized void formation, accelerating dendrite initiation (Figure 6b). By acting as an interlayer, the DA coating suppresses localized growth and stabilizes the Li|LPSCl interface. We further observed that LPSCl coated with 2 wt% DA exhibits a lower porosity (6.9%) compared to bare LPSCl (10.1%) (Figure S15). This echoes a recent study by Liu et al. [33], who reported that coating LPSCl with undecanethiol enhances SE pellet porosity, thereby reducing interparticle friction due to its soft nature. SEM images (Figure S16) collected for Li metal recovered post‐cycling and peeled from the SE separators were also revealing. For bare LPSCl, the surface exhibits pronounced roughness, indicative of non‐uniform lithium deposition. In contrast, Li recovered from DA–LPSCl showed a comparatively smoother surface. While SEM primarily provides morphological information, these observations suggest differences in deposition behavior, with the denser morphology generally associated with more uniform lithium deposition. We suggest that these factors contribute to the better performance of DA–LPSCl in Li symmetric cells.

Building on these insights, we further assembled full cells with Li metal anodes, rather than Li–In. The long‐term cycling performance of DA–LPSCl and bare LPSCl ASSBs with a Li metal anode is shown in Figure 6c,d, at a moderate CAM loading (∼7 mg cm^−2^). The DA–LPSCl cell maintained a good capacity of 166 mAh.g^−1^ after 100 cycles at 0.2 C (94% capacity retention) and 143 mAh.g^−1^ after 300 cycles (81% capacity retention). By contrast, the bare LPSCl cell delivered only 97 mAh.g^−1^ after 100 cycles, corresponding to only 57% capacity retention, and suffered from rapid capacity decay with a poor average CE of ∼90% during the first 20 cycles. This persistently low CE likely originates from poor interfacial contact between the NCM cathode and LPSCl under the relatively modest pressure applied during cycling (35 MPa), which leads to void formation and continuous side reactions. This delays the establishment of a stable interphase and sustains parasitic reactions over multiple cycles. At higher stack pressures, these voids are mechanically collapsed, enabling intimate contact after the first cycle and causing the CE to rise sharply as uniform current distribution suppresses side reactions. Notably, this strong pressure dependence is largely mitigated in the DA–LPSCl composite cathode. The mechanical softness and conformability of the DA layer promote better particle–particle contact within the composite cathode, which effectively infiltrates and bridges nanoscale voids at the interfaces, thereby minimizing the need for high external stack pressure to achieve intimate contact (Figure 6e).

To our knowledge, reports on solid‐state full‐cell cycling employing LPSCl as the SE in combination with a Li metal anode remain relatively limited. Nevertheless, the electrochemical performance achieved in this work compares favorably with representative prior studies that employ interfacial or electrolyte modification strategies to stabilize LPSCl‐based full cells. For example, Luo et al. [35] reported that a g‐C_3_N_4_ coating on LPSCl led to improved electrochemical performance relative to the uncoated electrolyte; the corresponding full cells exhibited good reversible capacities under their reported conditions but required additional coating layers (LiNbO_3_) on the CAM. Similar to our study, Liu et al. [11] demonstrated enhanced cycling stability using NCM811 and Li metal with 1‐undecanethiol‐modified LPSCl (UDSH@LPSCl), achieving a discharge capacity of 160 mAh g^−^^1^ with 80% capacity retention after 50 cycles at an areal capacity of 1.5 mAh cm^−^^2^. Hood et al. [29] placed an Al_2_O_3_ coating on LPSCl by atomic layer deposition, which suppressed chemical reactivity at the Li–SE interface. However, no full‐cell data were presented, nor was the effect of reactivity suppression on the cathode side evaluated. Kim et al. [37] coated LPSCl with polydimethylsiloxane (PDMS) or fluorinated PDMS and demonstrated stable cycling, although only up to 50 cycles and with limited improvement compared to the cell using bare LPSCl. Within this context, the present study demonstrates competitive full‐cell capacity and cycling stability under comparable or more demanding conditions, while relying on a distinct and comparatively simple interfacial modification strategy.

## 3 Conclusions

In this study, we demonstrate a simple and scalable organic coating strategy that addresses the critical limitations of sulfide SEs. By applying a conformal DA coating to LPSCl, we achieve a significant improvement in moisture tolerance compared to bare LPSCl and retain ionic conductivity above 1 mS cm^−^^1^ after exposure to 39% relative humidity for 2 h. This enhancement in air stability allows processing of the sulfide SEs under less stringent conditions, reducing reliance on costly ultra‐low‐humidity dry rooms. Importantly, this organic coating approach avoids the high‐temperature annealing and complex processing associated with conventional inorganic coatings, making it highly cost‐effective and compatible with existing battery manufacturing infrastructure. Furthermore, the DA coating provides dual protection at both the cathode and anode interfaces. On the cathode side, oxidative decomposition of LPSCl at high voltages is significantly suppressed, enabling stable cycling with Ni‐rich NCM cathodes and mitigating interfacial resistance growth. On the anode side, the DA–LPSCl electrolyte enables stable Li plating and stripping for over 1000 h in symmetric cells in comparison to the bare LPSCl, which short‐circuited in ∼230 h. Full cells employing DA–LPSCl as the catholyte and separator, Li metal as the anode, and Ni‐rich NCM85 as the cathode retained 81% of their initial capacity after 300 cycles at 0.2 C. Overall, this work establishes organic coatings on sulfide SE as a viable, low‐cost, and scalable approach to simultaneously enhance moisture tolerance, interfacial stability at both cathode and anode, and overall manufacturability. These findings pave the way for the practical realization of high‐energy, durable, and cost‐effective sulfide‐based ASSBs.

## Author Contributions

**Lanting Qian**: conceptualization, investigation, writing – original draft, methodology, validation, writing – review and editing, data curation, formal analysis, visualization. **Cameron Dean**: methodology, visualization, data curation, validation, investigation. **Ivan Kochetkov**: investigation, methodology, validation, visualization, data curation. **Hengning Chen**: investigation, data curation, methodology, validation. **Yangyang Huang**: data curation. **Linda Nazar**: conceptualization, funding acquisition, validation, writing – review and editing, project administration, visualization, supervision, resources.

## Funding

This work was supported by the Ontario Research Foundation and the Natural Sciences and Engineering Research Council of Canada.

## Conflicts of Interest

The authors declare no conflicts of interest.

## Supporting information




**Supporting File**: anie73015‐sup‐0001‐SuppMat.docx.

## Data Availability

The data that support the findings of this study are available in the Supporting Information of this article.
