# Supplementary material for: Enabling Moisture and Interfacial Stability in Sulfide Solid Electrolytes via a Processable Organic Coating Strategy for High‐Voltage All‐Solid‐State Batteries
Source: Angew Chem Int Ed Engl. 2026 Jun 30;65(34):e9983580. doi: 10.1002/anie.9983580 (PMC13480750; doi:10.1002/anie.9983580)
Supplement: Supplementary file 1 [file ANIE-65-e9983580-s001.docx]

***Supporting Information for:***

**Enabling Moisture and Interfacial Stability in Sulfide Solid Electrolytes via a Processable Organic Coating Strategy for High-Voltage All-Solid-State Batteries**

*Lanting Qian, Cameron Dean, Ivan Kochetkov, Hengning Chen, Yangyang Huang, and Linda Nazar**

Department of Chemistry, Waterloo Institute of Nanotechnology, University of Waterloo, Ontario, N2L 3G1, Canada.

**Methods**

***Synthesis of decanoic acid coated-LPSCl and evaluation of moisture stability***

Decanoic acid (99.5%) was purchased from MSE Supplies LLC, and LiNi_0.85_Mn_0.1_Co_0.05_O_2_ (NMC85, D_50_ ~ 4 µm) was obtained from BASF. Under an argon atmosphere, decanoic acid was dissolved in toluene and stirred for 3 hours to ensure complete dissolution. LPSCl was then added to the solution and stirred overnight. The solution was subsequently evaporated at 140 °C under a controlled nitrogen flow while being stirred at 500 rpm. The resulting powder was dried at 120 °C for 12 hours in a BUCHI glass oven connected to a vacuum line to remove residual toluene. After moisture exposure, the pellets were dried at 80 °C in a BUCHI glass oven, transferred to an argon-filled glovebox, reground, and subsequently characterized by ionic conductivity measurements to evaluate the effects of moisture exposure.

***Powder X-ray Diffraction***

Powder X-ray Diffraction (XRD) measurements on all materials were conducted at room temperature on a PANalytical Empyrean diffractometer equipped with a Pixcel bidimensional detector using Cu–Kα radiation. To examine structural changes before and after DA coating (**Fig. S2**), bare LPSCl and DA-LPSCl powders were spread on a zero-background sample holder in a glove box and sealed with Kapton film and vacuum grease to protect them from air exposure during data acquisition. The air stability of bare LPSCl and DA-LPSCl pellets was assessed by exposing the samples to ambient relative humidity (~39%) for varying durations; XRD measurements for these samples were conducted without Kapton sealing.

***Microscopy Measurements and Focused Ion Beam Milling***

Scanning electron microscopy images of the coated cathode were acquired using a Zeiss field emission scanning electron microscope. Cryogenic transmission electron microscopy images were collected with a Titan 80-300 LB microscope. FFT transformation, image process, and d-spacing were measured using GMS Digital Micrograph software.

***X-ray Photoelectron Microscopy and Time-of-Flight Secondary Ion Mass Spectrometry***

XPS was performed using a Thermo Fisher K-Alpha X-ray spectrometer with dual turbo molecular pumps and a monochromated, micro-focused, low-power Al K-Alpha X-ray source with a 128-channel detector to enable rapid acquisition. ToF-SIMS spectra were acquired using an ION-TOF 5 instrument in negative-ion or positive-ion mode with a 30 keV cluster primary ion gun over an area of 200 × 200 μm^2^, and 256 × 256 pixels were used to raster this area. The principal ion current was approximately 0.3 pA. The stop condition was set to 5 × 1012 ions per cm^2^ to enable semiquantitative analysis. Data evaluation was conducted using SurfaceLab v.7.2 software (IONTOF). Secondary-ion signals were normalized to the total ion signal, with all intensity data collected from the corresponding normalized secondary-ion images to ensure accurate relative intensity measurements. The relatively large analysis area, encompassing numerous SE-NCM85 interfaces and multiple NCM85 particles (BASF, D₅₀ ≈ 5 µm), was selected to minimize sampling bias.

***Cell assembly and battery cycling***

All-solid-state batteries were assembled in an argon-filled glovebox using commercial (Ampcera) or decanoic acid-coated LPSCl as the solid-state electrolyte, and either a LiIn alloy or Li metal anode. Uncoated NCM85 was used as the cathode. A PEEK cylinder was first filled with 120 mg of DA-LPSCl or bare LPSCl powder and pressed at 250 MPa for 1 minute to form the electrolyte separator pellet. Composite cathodes were prepared by grinding the argyrodite and bare NMC85 in an agate mortar at a weight ratio of 2:8 for approximately 15 minutes. The composite cathode was then evenly spread and pressed onto the SE pellet at 2 tons for ~3 minutes. On the opposite side of the pellet, a 10 mm-diameter, 0.1 mm-thick indium foil (CMR-direct) was applied, followed by ~1.5 mg of Li metal (Sigma-Aldrich) pre-flattened on copper foil to form the LiIn alloy anode. Alternatively, lithium metal foils were punched and polished with a clean blade to ensure surface smoothness and remove any surface contaminants. A custom-made cell casing was used to apply an initial pressure of ~35 MPa prior to sealing the assembly in an air-tight environment. Galvanostatic cycling was conducted using a VMP3 (Bio-Logic) or MACCOR cycler, with cycling parameters (voltage range and rate) detailed in the corresponding figures (1C = 200 mA·g⁻¹ for NMC85). All cycling was performed at room temperature (25 °C). Electrochemical impedance spectroscopy (EIS) was performed at 25 °C using a Bio-Logic SP-200 potentiostat over a frequency range of 100 mHz to 1 MHz. EIS data fitting was carried out using RelaxIS software.

***Cyclic voltammetry (CV)***

100 mg of solid SE-carbon nanofiber composite electrolyte was placed into a PEEK cylinder and compressed at 250 MPa for 1 minute to form a pellet with a 10 mm diameter for CV measurements. Subsequently, 50 mg of solid electrolyte powder was evenly distributed over one side of the LPSCl pellet and pressed at 250 MPa for an additional minute. The SE-carbon nanofiber composite electrode was prepared by mixing bare LPSCl or DA-LPSCl and carbon nanofiber in a weight ratio of 95:5, followed by hand-grinding the mixture in an agate mortar for 20 minutes. The Li-In electrode was fabricated in a similar manner described above for cell assembly. Finally, a load cell was employed to measure the initial 200 MPa applied pressure before enclosing the entire assembly in a stainless-steel casing. The CV measurements were performed using a VMP3 potentiostat (Bio-Logic) at a scan rate of 0.1 mV·s^-1^.

***Theoretical Calculations***

***Molecule Conformations***

Since the bulk structure of the DA surface coating is unknown, we began by characterizing the isolated DA molecules. First, we obtained the 10 most favourable structure conformations from (1) and calculated their ground-state energies using density functional theory (DFT) as implemented in Gaussian 16e^2^. Following recommendations from (3), we employed Dunning’s correlation-consistent quadruple-zeta basis set^4,5^ with Grimme’s D3 empirical dispersion corrections^6^ selected for calculations. Quadruple-zeta was selected for the basis set size, and it was augmented with diffuse functions to ensure accuracy. The atomic simulation environment (ASE)^7^ was used to create input files for these calculations. The resulting conformation energies (**Table S1**) were used to calculate the probability of each conformation at room temperature under the Boltzmann distribution at room temperature. The partition function for the system is shown in **Equation S1**, and the probability calculation is shown in **Equation S2**.

$Z = \sum_{i} e^{\frac{-E_{i}}{k_{B}T}}$	(**Eqn.** **S1**)

$P(E_{i}) = \frac{e^{\frac{-E_{i}}{k_{B}T}}}{Z}$	(**Eqn.** **S2**)

Conformation #1 was found to be present in the highest proportion for isolated DA molecules, with a probability of ~70 %. This conformation corresponds to the linear orientation of the molecule where the carboxylic acid and CH_3_ terminations have a maximum distance of separation. This linear conformation with the lowest energy was selected subsequently and used for all surface adsorption calculations.

***Surface Selection***

We performed density functional theory (DFT) calculations to identify the most stable argyrodite surfaces. For these calculations, we used the Vienna *ab initio* simulation package (VASP)^8,9^ to perform ionic relaxation on symmetric, low-index surfaces. The calculation employed the projector augmented wave (PAW) method, which represents electron orbitals using plane waves and approximates core electrons using pseudopotentials. The exchange-correlation energy functional was treated under the Purdue-Burke-Ernzerhof (PBE) version of the generalized gradient approximation (GGA)^10,11^. These calculations used a plane wave cutoff of 520 eV, and a Monkhorst-Pack k-point mesh was applied, with a density of at least 300/N, where N is the number of atoms. The total energy was converged to at least 10^-5^ eV, and the forces were converged to 0.01 eV/Å. The initial structure of argyrodite (mp-985592) was obtained from the Materials Project Database.^12^ The Python Materials Genomics (pymatgen) package was used to generate surface slabs data and VASP input files^13^. A 10 Å vacuum was used on both side of the surface to prevent spurious periodic interactions. As seen in **Table S2**, one of the sulfur-poor 010 index planes exhibited the lowest surface energy and was selected for further calculations.

***Binding Energy Calculations***

Binding energy calculations were carried out by then placing the DA molecule at various locations and orientations on the surface of LPSCl. To select the absorption positions, five distinct surface atoms on LPSCl were chosen as initial binding sites. These sites were selected to ensure a wide spatial distribution along the surface and included at least one of each element closest to the surface.

VASP was used to calculate the binding energies of DA molecules in each of the configurations outlined above. The same relaxation parameters were used as the argyrodite surface relaxations, except for an increased 15 Å vacuum on the side where the molecule was deposited. 10 configurations were simulated, combining 5 surface sites with 2 orientations of the molecules. The molecules were initially placed with a gap of 2 Å from the surface, with either the polar head or nonpolar tail interacting with the surface atoms. This perpendicular orientation was selected for computational tractability, since the number of simulated atoms would need to increase significantly to accommodate a molecule parallel to the surface while still maintaining a 10 Å gap to prevent molecule-molecule interaction. Nonetheless, interactions of CH_2_ groups along the chain are expected to be similar in nature to the interaction of the -CH_3_ tail. Therefore, the comparison between the -COOH group and -CH_3_ tail is sufficient. No constraints were used to prevent the motion of atoms on the opposite face of the slab, as **Figure S2** revealed that these atoms were stationary throughout the relaxation. This indicates that the slab thickness and vacuum size were large enough to prevent interaction between the binding site atoms and the opposite face of the slab. The binding energy for each configuration was calculated using **Equation S3**.

$Binding Energy = E(deposited DA) - E(surface) - E(DA molecule)$	(**Eqn.** **S3**)

**List of Supporting Figures**

**Figure S1.** SEM images of bare LPSCl and DA-LPSCl.

**Figure S2.** XRD patterns of bare LPSCl and DA-LPSCl measured using Kapton tape to protect the samples.

**Figure S3.** The most stable surface of argyrodite (LPSCl) calculated from DFT, corresponding to the (010) plane with a surface energy of 0.4 J m⁻².

**Figure S4.** Visualization of different DA conformations after structural relaxation.

**Figure S5.** Visualization of selected binding sites on LPSCl.

**Figure S6.** Stationary bottom surface of LPSCl. No constraints were used to prevent the motion of atoms on the opposite face of the slab, showing that these atoms were stationary throughout the relaxation. This indicates that the slab thickness and vacuum size were large enough to prevent interaction between the binding site atoms and the opposite face of the slab.

**Figure S7.** P *2p* X-ray photoelectron spectra (taken with transfer to the XPS in an inert-atmosphere transfer chamber) on pressed pellets of a) bare and b) 2 wt% DA-LPSCl.

**Figure S8.** Charge and discharge profiles of ASSBs using 5 wt% DA-LPSCl as the catholyte and separator, cycled at 0.2 C in the voltage window of 2.8 to 4.3 V versus Li^+^/Li. The cathode is NCM85 and the anode is Li-In.

**Figure S9.** Cycling performance of a cell assembled with 2 wt% DA-LPSCl as the catholyte and separator, cycled at 0.2 C in the voltage window of 2.8-4.6 V versus Li^+^/Li. The cathode is NCM85 and the anode is Li-In.

**Figure S10.** Cycling battery performance of a high-cathode loading (21 mg.cm^-2^) cell assembled with 2 wt % DA-LPSCl as the catholyte and separator, cycled at 0.2 C in the voltage window of 2.8 to 4.3 V versus Li^+^/Li. The cathode is NCM85 and the anode is Li-In.

**Figure S11.** Cyclic voltammograms (CVs) of DA-LPSCl and LPSCl solid electrolytes in carbon-SE||In cells scanned from OCV to 5.0 V followed by a reverse scan and return to OCV at 0.1 mV s^−1^. a) comparison of the initial cycle of bare LPSCl and 2 wt% DA-LPSCl; CV curves from the 1^st^ to 3^rd^ cycle for b) bare LPSCl and c) 2 wt% DA-LPSCl.

**Figure S12.** Transmission line model used for evaluating the impedance growth in the composite cathode.

**Figure S13.** FIB cross-sectional images of DA-LPSCl after 150 cycles at a 0.2C rate. ASSBs were assembled using the DA-LPSCl or bare LPSCl as the catholyte and separator, and Li-In as the anode at a pressure of 200 MPa.

**Figure S14.** FIB cross-sectional images of bare LPSCl after 150 cycles at a 0.2C rate. ASSBs were assembled using the DA-LPSCl or bare LPSCl as the catholyte and separator, and Li-In as the anode at a pressure of 200 MPa.

**Figure S15.** Relative densities and corresponding porosities of bare LPSCl, 2 wt% DA–LPSCl, and 5 wt% DA-LPSCl pellets, highlighting the progressive densification (reduced porosity) upon adding more DA.

**Figure S16.** SEM images comparing the lithium metal surface morphology after cycling in symmetric cells using DA-LPSCl (left column) and bare LPSCl (right column).

**List of Tables**

**Table S1**. Conformation energies and probabilities associated with DA adopting each conformation

| Conformation Number | Energy Relative to Lowest Energy Conformation [K] | Probability |
| --- | --- | --- |
| 1 | 0.0 | 69.8% |
| 2 | 4015.9 | 0.0% |
| 3 | 1072.2 | 1.8% |
| 4 | 4234.0 | 0.0% |
| 5 | 1628.3 | 0.3% |
| 6 | 1411.9 | 0.6% |
| 7 | 8114.8 | 0.0% |
| 8 | 274.9 | 27.3% |
| 9 | 1715.5 | 0.2% |
| 10 | 4479.1 | 0.0% |

**Table S2**. Argyrodite surface terminations and their calculated energies

| **Miller Indices** | **Surface Composition** | **Simulated Slab** | **Surface Energy**  **(J/m^2^)** |
| --- | --- | --- | --- |
| 010 | S | Li_37_P_8_S_35_Cl_11_ | 0.70 |
| 010 | Li | Li_41_P_8_S_35_Cl_11_ | 0.44 |
| **010** | **Li_5_SCl** | **Li_36_P_6_S_28_Cl_10_** | **0.40** |
| 010 | Li_7_SCl | Li_40_P_6_S_28_Cl_10_ | 0.72 |
| 011 | Li_4_S_2_ | Li_32_P_6_S_26_Cl_10_ | 0.48 |
| 011 | Li_4_S_2_ | Li_34_P_6_S_28_Cl_8_ | 0.70 |

**Table S3**. Mass loading and ionic conductivity of DA-LPSCl as a function of decanoate content (wt%).

| **DA wt% added (%)** | Ionic conductivity (mS.cm^-1^) at 26 ± 1 °C |
| --- | --- |
| 1 | 1.4 |
| 2 | 1.3 |
| 5 | 0.7 |
| 10 | 0.2 |

**Table S4**. Comparison of reported surface-modified LPSCl solid electrolytes under various air exposure conditions, including exposure time and relative humidity (RH).

| Material | Air exposure time | Relative humidity (%) | Conductivity after coating (mS cm^-1^) | Conductivity retention (%) | Reference |
| --- | --- | --- | --- | --- | --- |
| OScA-LPSCl | 30 minutes | 35% | 3.02 | 83 | 15 |
| Li_6.03_P_1-x_Se_0.03_S_5_Cl | 30 minutes | 10% | 3.06 | 57 | 16 |
| ZSM-5 zeolite-LPSCl | 1 hour | 50% | 1.27 | 31 | 17 |
| LPSBI-Bromo | 2 hours | 0.5% | 2.8 | 89 | 18 |
| UDSH@LPSC | 5 hours | 33% | 2.1 | 86 | 19 |
| UDSH@LPSC | 3 days | 33% | 2.1 | 38 | 19 |
| OPA-LPSClBr | 24 hours | <1% RH | 2.5 | 92 | 20 |
| g-C_3_N_4_-LPSCl | 3 hours | 20% | 0.6 | 72 | 21 |
| PBS@LPSCl | 1 hour | 50% | N/A | ~90 | 22 |
| F-PDMS@LPSCl | 72 hours | <1% RH | 2.0 | 49 | 23 |
| Al_2_O_3_@LPSCl | 4 hours | Dry O_2_ exposure | 1.7 | N/A | 24 |
| DA-LPSCl | 2 hours | 39% | 1.13 | 91% | This work |

*OScA: oxysulfide-coated LPSCl. LPSBI: Li_7_P_2_S_8_Br_0.5_I_0.5_. Bromo:1-bromopentane or 1BR, C_5_H_11_Br. LPSClBr: Li_6_PS_5_Cl_0.5_Br_0.5_. UDSH: undecanethiol. OPA: phosphonic acid. PBS: polyborosiloxane. F-PDMS: fluorinated polydimethylsiloxane.

**References**

1. *PubChem*. Capric Acid. pubchem.ncbi.nlm.nih.gov. <https://pubchem.ncbi.nlm.nih.gov/compound/Capric-Acid>.

2. M. J. Frisch, G. W. Trucks, H. B.Schlegel, G. E. Scuseria, M. A. Robb, J. R. Cheeseman, J. A.Montgomery, Jr., T. Vreven, K. N. Kudin, J. C. Burant, J. M.Millam, S. S. Iyengar, J. Tomasi, V. Barone, B. Mennucci, M.Cossi, G. Scalmani, N. Rega, G.A. Petersson, H. Nakatsuji, M. Hada, M. Ehara, K. Toyota, R. Fukuda, J. Hasegawa, M. Ishida,T. Nakajima, Y. Honda, O. Kitao, H. Nakai, M. Klene, X. Li, J. E. Knox, H. P. Hratchian, J. B. Cross, V. Bakken, C. Adamo, J. Jaramillo, R. Gomperts, R. E. Stratmann, O. Yazyev, A. J.Austin, R. Cammi, C. Pomelli, J. W. Ochterski, P. Y. Ayala, K.Morokuma, G. A. Voth, P. Salvador, J. J. Dannenberg, V. G. Zakrzewski, S. Dapprich, A. D. Daniels, M. C. Strain, O. Farkas,D. K. Malick, A. D. Rabuck, K. Raghavachari, J. B. Foresman, J. V. Ortiz, Q. Cui, A. G. Baboul, S. Clifford, J. Cioslowski, B. B. Stefanov, G. Liu, A. Liashenko, P. Piskorz, I. Komaromi, R. L. Martin, D. J. Fox, T. Keith, M. A. Al-Laham, C. Y. Peng, A. Nanayakkara, M. Challacombe, P. M. W. Gill, B. Johnson, W. Chen, M. W. Wong, C. Gonzalez, and J. A. Pople, Gaussian, Inc.,Wallingford CT. Revision C.01. Gaussian, Inc. **2016.**

3. M. Bursch, J. Mewes, A. Hansen, S. Grimme, Best‐Practice DFT Protocols for Basic Molecular Computational Chemistry**. *Angew. Chemie. Int. Ed.* **2022**, *61*, e202205735.

4. Dunning. T. H, Gaussian Basis Sets for Use in Correlated Molecular Calculations. I. The Atoms Boron through Neon and Hydrogen. *J. Chem. Phys.* **1989**, *90*, 1007–1023.

5. R. A. Kendall, T. H. Dunning, R. J. Harrison, Electron Affinities of the First‐Row Atoms Revisited. Systematic Basis Sets and Wave Functions. *J. Chem. Phys.* **1992**, 96, 6796–6806.

6. S. Grimme, J. Antony, S. Ehrlich, H. A. Krieg, Consistent and Accurate Ab Initio Parametrization of Density Functional Dispersion Correction (DFT-D) for the 94 Elements H-Pu. *J. Chem. Phys*. **2010**, *132*, 154104.

7. A.H. Larsen, J.J. Mortensen, J. Blomqvist, I. E. Castelli, R. Christensen, M. Dułak, J. Friis, M. N. Groves, B. Hammer, C. Hargus, E. D. Hermes, P. C. Jennings, P. B. Jensen, J. Kermode. J. R. Kitchin, E. L. Kolsbjerg, J. Kubal, K. Kaasbjerg, S. Lysgaard, J. B. Maronsson, The Atomic Simulation Environment—a Python Library for Working with Atoms. *J. Phys. Condens. Matter* **2017**, *29*, 273002.

8. G. Kresse, J. Hafner, Ab Initio molecular Dynamics for Liquid Metals. *Phys. Rev. B* **1993**, *47*, 558–561.

9. G. Kresse, J. Furthmüller, Efficiency of Ab-Initio Total Energy Calculations for Metals and Semiconductors Using a Plane-Wave Basis Set. *Comput. Mater. Sci.* **1996**, *6*, 15–50.

10. P. E. Blöchl, Projector Augmented-Wave Method. *Physical Review B* **1994**, *50*, 17953–17979.

11. J. P. Perdew, K. Burke, M. Ernzerhof, Generalized Gradient Approximation Made Simple. *Phys. Rev. Lett.* **1996**, *77*, 3865–3868.

12. A. Jain, S. P. Ong, G. Hautier, W. Chen, W. D. Richards, S. Dacek, S. Cholia, D. Gunter, D. Skinner, G. Ceder, K.A. Persson, Commentary: The Materials Project: A Materials Genome Approach to Accelerating Materials Innovation*. APL Mater* **2013**, *1*, 011002.

13. S.P. Ong, W. D. Richards, A. Jain, G. Hautier, M. Kocher, S. Cholia, D. Gunter, V. L. Chevrier, K. A. Persson, G. Ceder, Python Materials Genomics (Pymatgen): A Robust, Open-Source Python Library for Materials Analysis. *Comp.Mater. Sci* **2013**, *68*, 314–319.

14. W.D. Jung, M. Jeon, S.S. Shin, J.S. Kim, H.G. Jung, B.K. Kim, J.H. Lee, Y.C. Chung, H. Kim, Functionalized Sulfide Solid Electrolyte with Air-Stable and Chemical-Resistant Oxysulfide Nanolayer for All-Solid-State Batteries. *ACS Omega*, **2020**, *40*, 26015-26022.

15. H.M. Kim, Y. Subramanian, K.S. Ryu, Improved Electrochemical and Air Stability Performance of SeS_2_ Doped Argyrodite Lithium Superionic Conductors for All-Solid-State Lithium Batteries. *Electrochim. Acta.* **2023**, *442*, 141869.

16. D. Lee, K.H. Park, Critical Role of Zeolites as H2S Scavengers in Argyrodite Li6PS5Cl Solid Electrolytes for All-Solid-State Batteries. *J. Mater. Chem. A.* **2021**, *9*, 17311-17316.

17. Z. Yu, S.L. Shang, K. Ahn, D.T. Marty, R. Feng, M.H. Engelhard, Z.K. Liu, D. Lu, Enhancing Moisture Stability of Sulfide Solid-State Electrolytes by Reversible Amphipathic Molecular Coating. *ACS Appl. Mater. Interfaces*. **2022**, *14*, 32035-32042.

18. M. Liu, J.J. Hong, E. Sebti, K. Zhou, S. Wang, S. Feng, T. Pennebaker, Z. Hui, Q. Miao, E. Lu, N. Harpak, S. Yu, J. Zhou, J.W. Oh, M.S. Song, J. Luo, R.J. Clément, P. Liu. Surface molecular engineering to enable processing of sulfide solid electrolytes in humid ambient air. *Nat. Comm.* **2025**, *16*, 213.

19. L. Fadillah, L. Braks, J. Oh, M. Liu, H. Türk, D. Tisi, M. Mensi, M. Ceriotti, J.W. Choi, A. Coskun. Molecular Surface Engineering of Sulfide Electrolytes with Enhanced Humidity Tolerance for Robust Lithium Metal All-Solid-State Batteries. *Adv. Mater.* **2025**, *38*, e15013.

20. M. Luo, C. Wang, Y. Duan, X. Zhao, J. Wang, X. Sun. Surface Coating Enabling Sulfide Solid Electrolytes with Excellent Air Stability and Lithium Compatibility. *Energy.Environ. Mater.* **2024**, *7*, e12753.

21. Y. Hong, J.Y. Jung, Y.S. Byeon, S.J. Park, K.J. Kim, W. Cho, M.S. Park. Surface Engineering of Argyrodite-Li6PS5Cl Solid Electrolytes with a Polyborosiloxane Copolymer for All-Solid-State Batteries. *Chem. Eng. J.* **2025**, *515*, 163406.

22. K.T. Kim, J. Woo, Y.S. Kim, S. Sung, C. Park, C. Lee, Y.J. Park, H.W. Lee, K. Park, Y.S. Jung. Ultrathin Superhydrophobic Coatings for Air-Stable Inorganic Solid Electrolytes: Toward Dry Room Application for All-Solid-State Batteries. *Adv. Energy. Mater.* **2023**, *13*, 2301600.

23. Z.D. Hood, A.U. Mane, A. Sundar, S. Tepavcevic, P. Zapol, U.D. Eze, S.P. Adhikari, E. Lee, G.E. Sterbinsky, J.W. Elam, J.G. Connel, Multifunctional Coatings on Sulfide-Based Solid Electrolyte Powders with Enhanced Processability, Stability, and Performance for Solid-State Batteries. *Adv. Mater.* **2023**, *35*, 2300673.
